# Association Analysis of Gut Microbiota and Prognosis of Patients with Acute Ischemic Stroke in Basal Ganglia Region

**DOI:** 10.3390/microorganisms11112667

**Published:** 2023-10-30

**Authors:** Jiayu Shi, Yiting Zhao, Qionglei Chen, Xiaolan Liao, Jiaxin Chen, Huijia Xie, Jiaming Liu, Jing Sun, Songfang Chen

**Affiliations:** 1 Department of Geriatrics, The Second Affiliated Hospital and Yuying Children’s Hospital of Wenzhou Medical University, Wenzhou 325027, China; lilichen41519@163.com (J.S.); 15867160109@163.com (Y.Z.); stepbystepchen44@163.com (Q.C.); dan21269171904@163.com (X.L.); rm120w@163.com (J.C.); xhj851@126.com (H.X.); 2 Department of Preventive Medicine, School of Public Health and Management, Wenzhou Medical University, Wenzhou 325035, China; wzjiaming_liu@163.com; 3 Department of Neurology, The Second Affiliated Hospital and Yuying Children’s Hospital of Wenzhou Medical University, Wenzhou 325027, China

**Keywords:** ischemic stroke, basal ganglia region infarction, gut microbiota, functional outcome, ROC curve

## Abstract

Previous studies have implied the potential impact of gut microbiota on acute ischemic stroke (AIS), but the relationships of gut microbiota with basal ganglia region infarction (BGRI) and the predictive power of gut microbiota in BGRI prognosis is unclear. The aim of this study was to ascertain characteristic taxa of BGRI patients with different functional outcomes and identify their predictive value. Fecal samples of 65 BGRI patients were collected at admission and analyzed with 16s rRNA gene sequencing. Three-month functional outcomes of BGRI were evaluated using modified Rankin Scale (mRS), and patients with mRS score of 0–1 were assigned to good-BGRI group while others were assigned to poor-BGRI group. We further identified characteristic microbiota using linear discriminant analysis effect size, and receiver operating characteristic (ROC) curve was used to determine the predictive value of differential bacteria. According to the mRS score assessed after 3 months of stroke onset, 22 patients were assigned to poor-BGRI group, while 43 patients were assigned to good-BGRI group. Short chain fatty acids-producing bacteria, *Romboutsia* and *Fusicatenibacter*, were characteristic microbiota of the good-BGRI group, while pro-inflammatory taxa, *Acetanaerobacterium*, were characteristic microbiota of the poor-BGRI group. Furthermore, the differential bacteria showed extensive associations with clinical indices. ROC curves, separately plotted based on *Romboutsia* and *Fusicatenibacter*, achieved area under the curve values of 0.7193 and 0.6839, respectively. This study identified the efficient discriminative power of characteristic microbiota in BGRI patients with different outcomes and provided novel insights into the associations of gut microbiota with related risk factors.

## 1. Introduction

Acute ischemic stroke (AIS), the most common subtype of stroke, is a main cause of disability and death worldwide [1,2]. The ischemic injury in different brain regions is correlated with different symptoms and outcomes. Wherein, basal ganglia region, referred to as the sub-cortical structures crucial for sensorimotor function [3], is specifically vulnerable to ischemic damage, in consequence, constituting the majority of silent brain infarcts [4]. Compared to other recent subcortical infarcts, patients with basal ganglia region infarction (BGRI) were more likely to have a larger infarct size [5]. Except for the conventional sensorimotor function of basal ganglia region, an increasing number of studies have revealed the potential relationships between BGRI with post-stroke fatigue [6] and cognitive impairment [7]. Due to the severe injury and more extensive complications caused by BGRI, recognizing adverse functional outcomes and intervening as early as possible are crucial to regain the ability to live independently for an individual. Up to date, several factors, including age [8], sex [9], onset time [10], stroke history [11], and National Institutes of Health Stroke Scale (NIHSS) score [12], are commonly used to evaluate the prognosis of AIS including BGRI. However, the accurate and convenient predictive biomarkers of prognosis of BGRI remain an important unmet clinical need.

Recently, an increasing number of studies have highlighted the importance of gut microbiota in AIS. Compared to healthy individuals, the ischemic stroke patients showed gut microbiota dysbiosis that was characterized by a deficiency of short chain fatty acids (SCFAs)-producing bacteria [13]. In another study, the butyrate-producing bacterium, *Roseburia*, was identified as it was pronouncedly and negatively correlated with dynamic changes in NIHSS score and modified Rankin Scale (mRS) score assessed at 30 days or 1 year after stroke onset [14]. In animal models, shaped by age [15] and gender [16], gut microbiota affected the outcome of AIS by neuroinflammation, and the attenuation of AIS mediated by Chinese medicinal formulae was attributed to altered gut microbiota and decreased inflammation levels [17]. Moreover, the ability of gut microbiota to discriminate disease states had been proved in various neuropsychological diseases. The model constructed based on the altered gut microbiota could discriminate epilepsy patients from healthy individuals [18]. Differential gut microbiota, coupled with nutritional data, were applied to distinguish patients diagnosed with Parkinson’s disease (PD) from healthy individuals [19]. Our previous study has also identified characteristic taxa to predict the functional outcome of AIS patients with hyperlipemia [20]. Notably, the effect of gut microbiota on the brain was regionally selective. The extent of structural–functional connectivity coupling in the fusiform gyrus, the inferior occipital, and the medial superior frontal gyrus were the targets of gut microbiota for cognitional impairment in healthy young adults [21]. In a rat experiment, *Lachnospiraceae* and *Ruminoccoceae* affected their social behaviors by altering their gene expression, especially in the prefrontal cortex [22]. However, no attention was paid to the associations of diverse ischemic regions and gut microbiota in previous studies.

In this study, we only focused on patients with BGRI to avoid discrepancy of gut microbiota caused by infarcts of different regions, and explored the altered structure and composition of gut microbiota between BGRI patients with good and poor outcomes. Furthermore, we focused on ascertaining the characteristic microbiota present in patients with a good outcome and identified the predictive value of differential bacteria for the prognosis of BGRI.

## 2. Materials and Methods

### 2.1. Patients Enrollment

A total of 114 patients with AIS associated with the basal ganglia region were recruited in the Department of Neurology, the Second Affiliated Hospital of Wenzhou Medical University from September 2020 to July 2021. These patients were all from Zhejiang Province. Inclusion criteria: (1) AIS patients with unilateral infarction of basal ganglia region (basal ganglia, internal capsule, and/or thalamus) [23,24] confirmed with magnetic resonance imaging (MRI), (2) admitted within 72 h of stroke onset, and (3) aged 40 to 90 years. AIS was diagnosed by neurologists according to the criteria illustrated by American Heart Association/American Stroke Association combined with brain computed tomography and MRI [25]. Exclusion criteria: (1) the presence of other cortical or subcortical strokes and prior infarcts or transient ischemic attack, (2) other brain abnormalities, (3) MRI contraindications, (4) stroke mimics, viz. epilepsy and migraine, (5) consumption of antibiotics or probiotics or self-reported dieting within 3 months, (6) patients with malignant tumors, a history of intestinal surgery, and severe intestinal diseases (e.g., inflammatory bowel disease and acute enteritis), and (7) other diseases affecting post-stroke functional outcomes (e.g., diabetic foot). Finally, 14 patients were excluded due to presence of bilateral cerebral hemisphere infarction, 35 patients were excluded according to exclusion criteria, and as a result, 65 patients with BGRI were enrolled. All patients were successfully followed up by telephone at 3 months after stroke onset, and mRS was evaluated to assess the post-stroke functional outcome of each patient. The good outcome group (good-BGRI group), was defined as one with an mRS score of 0–1, and the poor outcome group (poor-BGRI group) was defined as one with an mRS score of 2–6. 

The study was approved by the Medical Ethics Committee of the Second Affiliated Hospital of Wenzhou Medical University. Informed written consent of all participants was also obtained prior to entry into the study.

### 2.2. Image Acquisition

All patients enrolled in this study underwent MRI scans using 1.5-T scanners (GE Signa HDXT; GE Healthcare, Waukesha, WI, USA). The protocol included T1-weighted image (T1WI, TR/TE = 2000/20 ms; Th = 5 mm; gap = 1 mm; FOV = 230 × 230 mm^2^), T2-weighted image (T2WI, TR/TE = 3000/80 ms; Th = 5 mm; gap = 1 mm; FOV = 230 × 230 mm^2^), fluid-attenuated inversion recovery (FLAIR, TR/TE = 7000/160 ms; Th = 1 mm; FOV = 230 × 230 mm^2^), and diffusion-weighted imaging (DWI, TR/TE = 5700/75 ms; gap = 1 mm; FOV = 230 × 230 mm^2^; 2b values of 0 and 1000 s/mm^2^).

### 2.3. Demographics and Clinical Data Collection

Demographic information was collected, including sex, age, educational level, marriage status, diabetes, hypertension, and hyperlipidemia. Patients who were receiving relevant medications and had a self-reported disease history were also eligible. Baseline clinical data were collected, including systolic blood pressure (SBP), diastolic blood pressure (DBP), mean arterial pressure (MAP), C-reactive protein (CRP), hypersensitive C-reactive protein (hs-CRP), homocysteine (Hcy), triglycerides (TG), total cholesterol (TC), high-density lipoprotein (HDL), low-density lipoprotein (LDL), glycosylated hemoglobin (HbA1c), fasting blood glucose (FBG), vitamin B12, folate, free triiodothyronine (FT3), free tetraiodothyronine (FT4), thyroid-stimulating hormone (TSH), and uric acid. Blood samples were withdrawn within 24 h of admission and after a fast of 8–12 h. Stroke severity, anxiety, depression, sleep quality, and cognitive impairment were assessed at admission by neurologists based on the NIHSS, Hamilton Anxiety Scale (HAMA), Hamilton Depression Scale (HAMD), Pittsburgh Sleep Quality Index (PSQI), and Mini-Mental State Examination (MMSE).

### 2.4. Fecal Sample Collection and Gut Microbiota Analysis

Fresh fecal samples (200 mg) were collected within 1 week after admission and transferred to the laboratory immediately. Samples were placed in a labeled 2 mL sterile centrifuge tube and stored at −80 °C within 30 min of preparation. Microbial DNA was extracted with OMEGA-soil DNA Kit (Omega Bio-Tek, Norcross, GA, USA) according to manufacturer’s instructions. NanoDrop2000 UV-vis spectrophotometer (Thermo Scientific, Wilmington, NC, USA) was used to detect the concentration and purity of DNA. The DNA extraction was followed by the amplification of the V3–V4 hypervariable regions in 16s rRNA gene of gut microbiota. Next, the sequencing was conducted on Illumina MiSeq System (Illumina, San Diego, CA, USA) by Majorbio Biopharm Technology Co., Ltd. (Shanghai, China).

Alpha diversity, including Ace index and Shannon index, was analyzed using Wilcoxon rank sum test. Beta diversity was compared using principal coordinate analysis (PCoA) of Bray–Curtis test. Relative abundance at family and genus levels was also calculated to exhibit the different microbial communities between the two groups. Linear discriminant analysis (LDA) effect size (LEfSe) using Kruskal–Wallis test was performed to determine the characteristic taxa from phylum to genus levels, and an LDA score of >2 was considered as the threshold. Moreover, significantly different genera were also identified based on the relative abundance of community using Wilcoxon rank sum tests. The heatmap reflecting the correlation between different genera and clinical indicators was presented by calculating Spearman correlation coefficient. Finally, receiver operating characteristic (ROC) curves were plotted based on differential bacteria as well as clinical parameters, and the area under the curve (AUC) was utilized to confirm the predictive capacity of gut microbiota for the prognosis of BGRI.

### 2.5. Statistical Analysis

In this study, continuous variables were represented by mean ± standard deviation or median and quartile distance depending on whether they were normally distributed. Student’s *t*-test was used to compare the variables conforming to normal distribution, and rank sum test was used to compare them otherwise. Categorical variables were expressed as percentages and analyzed with chi-square test. Covariates, showing significant differences between two groups or known as risk factors for stroke outcome, were selected to build multivariate logistic regression model. Odds ratios (ORs) with 95% confidence interval (CI) were reported. *p* < 0.05 was considered significant. Statistical analysis was performed using SPSS V.22.0 (SPSS, Chicago, IL, USA), GraphPad Prism V.9.0.0 (La Jolla, CA, USA), and Adobe Illustrator 2022 (Adobe Systems Incorporated, San Jose, CA, USA).

## 3. Results

### 3.1. Baseline Characteristics of Patients

According to the 3-month follow-up, 43 patients with a favorable outcome and 22 patients with an unfavorable outcome were identified, and the demographics and clinical characteristics of all patients are summarized in Table 1. In the good-BGRI group, 53.5% were male, and the mean age was 63.4 years, while in poor-BGRI group, 45.5% were male, and the mean age was 67.1 years. Significantly higher hs-CRP (*p* = 0.005), NIHSS score (*p* = 0.001), HAMA score (*p* = 0.019), and HAMD score (*p* = 0.001) were observed in the poor-BGRI group. There were no significant differences in sex, age, marriage status, educational level, hypertension, diabetes, hyperlipemia, PSQI score, MMSE score, blood pressure, and other biochemical indicators between the two study groups. Furthermore, multivariate regression analysis, as shown in Table 2, adjusted covariates, including gender, age, hs-CRP, HbA1c, Hcy, TG, TC, LDL, NIHSS score, HAMA score, HAMD score, and PSQI score, revealed that Hcy (OR = 1.297, *p* = 0.029), NIHSS score (OR = 1.901, *p* = 0.023), and HAMD score (OR = 1.738, *p* = 0.046) were independent risk factors of the poor-BGRI group.

### 3.2. The Different Gut Microbiota Diversities between Good-BGRI Group and Poor-BGRI Group

**Figure 1:**
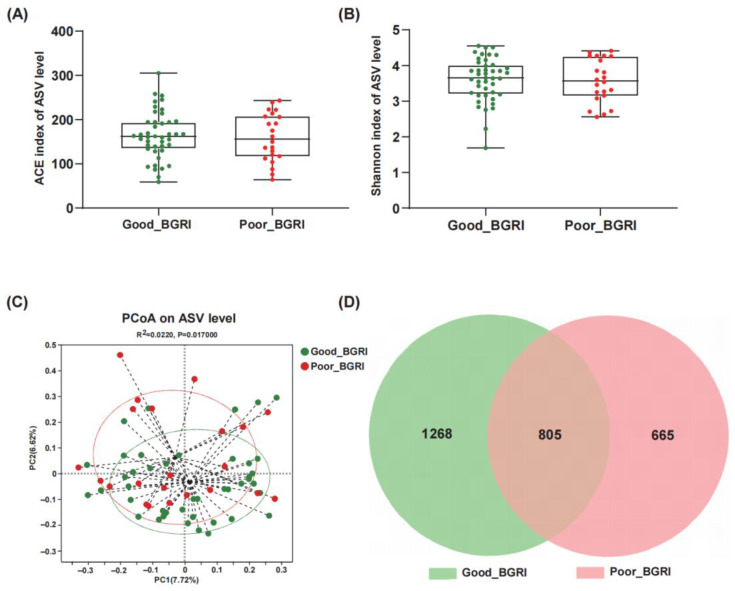
Different alpha and beta diversities of gut microbiota between good-BGRI group and poor-BGRI group. Alpha diversity was analyzed using Ace index (**A**) and Shannon index (**B**). Beta diversity was analyzed using PCoA of Bray–Curtis test (**C**). Venn diagram shows both unique and shared ASVs between the good- and poor-BGRI groups (**D**). The green circle represents the good-BGRI group, while the red circle represents the poor-BGRI group.

Alpha diversity was evaluated using Ace index and Shannon index. The good-BGRI group showed a slightly increasing trend both in Ace index (*p* = 0.6978, Figure 1A) and Shannon index (*p* = 0.6031, Figure 1B) in comparison with the poor-BGRI group. Additionally, beta diversity showed a remarkable difference between the two groups (Figure 1C) (Adonis R^2^ = 0.022, *p* = 0.017). Furthermore, the Venn diagram showed that 805 of the total 2738 ASVs were shared between the two groups, with 1268 and 665 ASVs unique for the good-BGRI group and the poor-BGRI group, respectively (Figure 1D). 

### 3.3. Different Compositions at Family and Genus Levels between Good-BGRI and Poor-BGRI Groups

**Figure 2:**
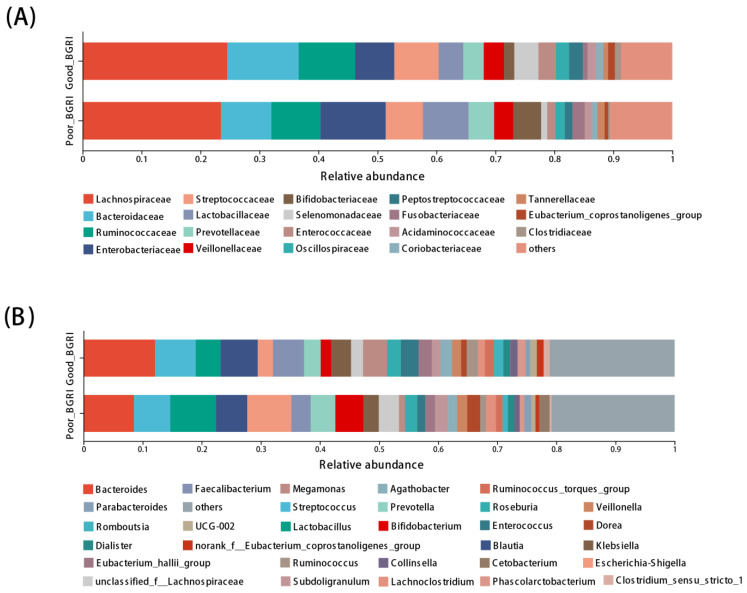
Different microbial compositions at family and genus levels between good-BGRI group and poor-BGRI group. The bar plots at family (**A**) and genus (**B**) levels showed relative abundance of gut microbiota between the good- and poor- BGRI groups. The color and width of grids indicate the relative abundance of different gut microbiota.

The top six dominant taxa at family level were consistent in the two groups, i.e., *Lachnosipraceae*, *Bacteroidaceae*, *Ruminococcaceae*, *Sterptococcaceae*, *Enterobacteriaceae*, and *Lactobacillaceae* (Figure 2A). At genus level, except for *Bacterioides*, the most abundant taxa in both the two groups, *Blautia*, *Faecalibacterium*, and *Enterococcus*, showed higher richness in the good-BGRI group, while the poor-BGRI group was more likely to have *Lactobacillus*, *Megamonas*, *Prevotella*, *Escherichia-Shigella*, *Bifidobacterium*, and *unclassified_f_Lachnospiraceae* (Figure 2B) in higher abundance.

### 3.4. The Differential Gut Microbiota in Good-BGRI Group

**Figure 3:**
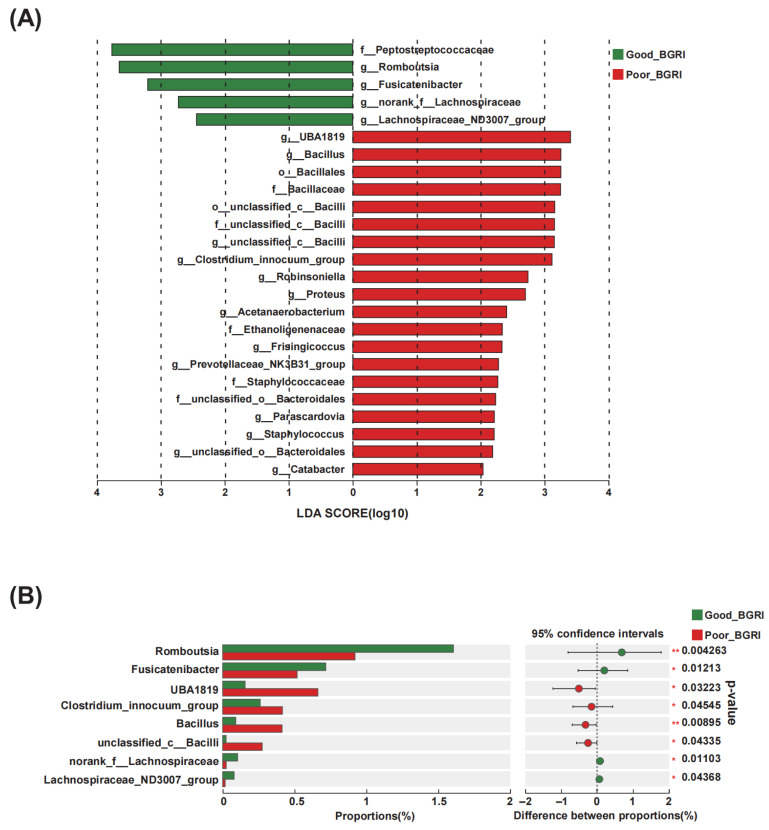
Significantly altered gut microbiota between good-BGRI group and poor-BGRI group. (**A**) LDA revealed differentially abundant taxa between the good-BGRI group and the poor-BGRI group from family to genus levels. The LDA scores (Log10) >2 are listed. (**B**) The bar plot shows the comparative analysis of several differential genera with the highest abundance between the good-BGRI group and the poor-BGRI group. * *p* < 0.05, ** *p* < 0.01.

To further identify the differential microbiota from phylum to genus levels, LEfSe analysis was conducted (Figure 3). At genus level, *Romboutsia*, *Fusicatenibacter*, *norank_f_Lachnospiraceae*, and *Lachnospiraceae_ND3007_group* were identified as differential genera in the good-BGRI group, while *UBA1819*, *Bacillus*, *unclassified_c_Bacilli*, *Clostridium_innocuum_group*, *Robinsoniella*, *Proteus*, *Acetanaerobacterium*, *Frisingicoccus*, *Prevotellaceae_NK3B31_group*, *Parascardovia*, *Staphylococcus*, *unclassified_o_Bacteroidales*, and *Catabacter* were identified in the poor-BGRI group (Figure 3A). Moreover, the top eight most abundantly characterized genera, namely, *Romboutsia*, *Fusicatenibacter*, *UBA1819*, *Clostridium_innocuum_group*, *Bacillus*, *unclassified_c_Bacilli*, *norank_f_Lachnospiraceae*, and *Lachnospiraceae_ND3007_group*, were also determined (Figure 3B).

### 3.5. Correlations of Gut Microbiota with mRS Scores and Clinical Indicators

**Figure 4:**
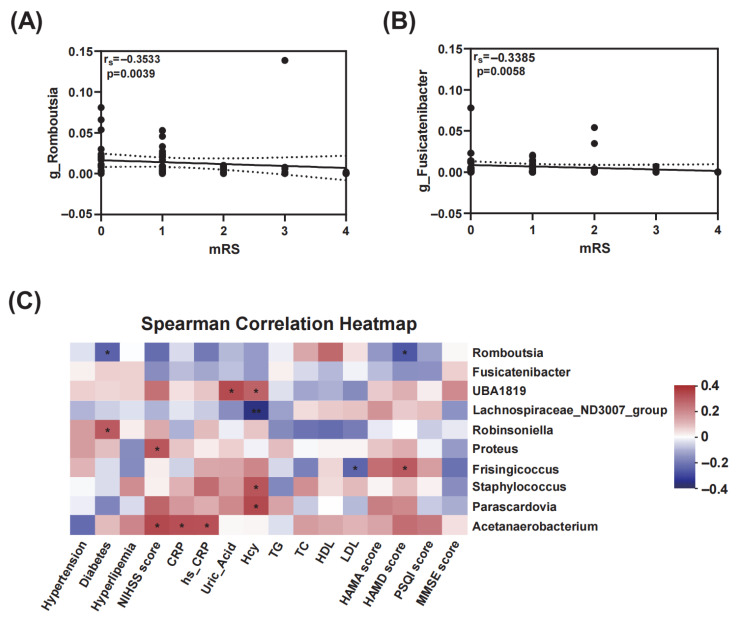
Correlations of gut microbiota with mRS scores and clinical indicators. The correlations of the relative abundance of *g_Romboutsia* (**A**) (*p* = 0.0039, rs = −0.3533) and *g_Fusicatenibacter* (**B**) (*p* = 0.0058, rs = −0.3385) with mRS were analyzed using Spearman’s rank correlation (rs) and probability (p). (**C**) Spearman correlation heatmap exhibited the relationships between taxa at genus level and clinical indicators as well as disease history. * *p* < 0.05, ** *p* < 0.01.

As shown in Figure 4A,B, *g_Romboutsia* (*p* = 0.0039) and *g_Fusicatenibacter* (*p* = 0.0058) showed significant negative correlation with mRS scores. Furthermore, the Spearman correlation heatmap revealed the correlations between characteristic gut microbiota and clinical indicators (Figure 4C). *Romboutsia* were negatively associated with diabetes and HAMD score. *UBA1819* showed a significantly higher abundance in patients with higher uric acid levels. Frisingicoccus was positively correlated with HAMD score. Additionally, NIHSS score was observed to have significantly positive correlations with *Proteus* and *Acetanaerobacterium*. Both CRP and hs-CRP had significant positive links with *Acetanaerobacterium*. Higher Hcy was observed in patients with higher abundance of *UBA1819*, *Staphylococcus*, and *Parascardovia*, while its relative abundance changed inversely with *Lachnospiraceae_ND3007_group*.

### 3.6. Predictive Performance of Differential Gut Microbiota from Good-BGRI Group and Clinical Parameters

**Figure 5:**
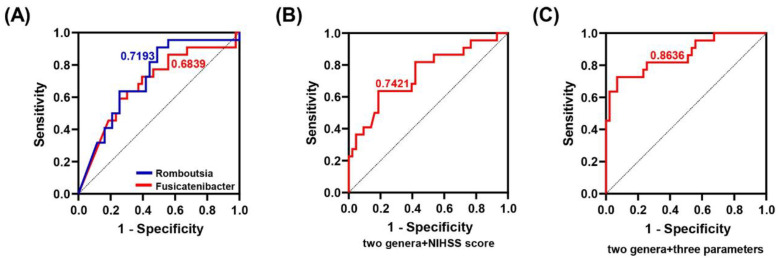
Performance of distinguishing good-BGRI group from poor-BGRI group based on good-BGRI group-related genera and clinical parameters. (**A**) *Romboutsia* and *Fusicatenibacter*, individually plotted, reached AUC values of 0.7193 (95% CI: 0.5901–0.8485) and 0.6839 (95% CI: 0.5433–0.8246), respectively. (**B**) Combination of these two genera and the NIHSS score resulted in an AUC value of 0.7421 (95% CI: 0.6102–0.8739). (**C**) Combination of these two genera and three parameters (NIHSS score, Hcy, and HAMD score) achieved an AUC value of 0.8636 (95% CI: 0.7638–0.9635).

ROC curve was used to further explore the predictive ability of characteristic microbiota as well as clinical indicators for the prognosis of BGRI. As shown in Figure 5A, *Romboutsia* and *Fusicatenibacter* were screened based on LDA values and achieved AUC values of 0.7193 (95% CI: 0.5901–0.8485) and 0.6839 (95% CI: 0.5433–0.8246), respectively. Further analysis exhibited AUC values of 0.7421 (95% CI: 0.6102–0.8739) and 0.8636 (95% CI: 0.7638–0.9635) based on the combination of the two genera and the NIHSS score as well as the combination of the two genera and three parameters (NIHSS score, Hcy, and HAMD score), respectively (Figure 5B,C). 

## 4. Discussion

This study explored the altered gut microbiota between the different functional outcomes of BGRI. As a result, we identified the characteristic microbiota in the good-BGRI group, viz., *Romboutsia* and *Fusicatenibacter*, and observed extensive correlations between the characteristic microbiota and stroke-related indices. Furthermore, ROC curves individually plotted based on these two genera all exhibited good sensitivity and specificity for prediction of BGRI prognosis. These results suggested that gut microbiota could act as accurate and convenient biomarkers to efficiently distinguish BGRI patients with high risk of poor functional outcome and further guide early intervention.

In the present study, NIHSS score, Hcy, and HAMD score were independent risk factors of the unfavorable functional outcome of BGRI. NIHSS is a traditional and widespread tool used to assess stroke severity with high reliability and predictive ability [26]. It exhibited an outstanding performance for early diagnosis compared to eight other prehospital stroke scales [27], and NIHSS score > 8, which was assessed within 24 h of mechanical thrombectomy, was an independent predictor of mRS 0–2 assessed 90 days after stroke onset [28]. As an independent risk factor of stroke [29], elevated Hcy was correlated with higher recurrence rate and the unfavorable functional outcome of stroke [30], especially in patients with atrial fibrillation [31]. In the mouse stroke model, higher Hcy level exaggerated brain damage by augmenting microglia activation and neuroinflammation [32], and a clinical trial revealed that lowering Hcy level decreased the risk of stroke [33]. In addition, it was reported that more severe pre-stroke depressive symptoms were associated with poorer functional and cognitive outcomes of stroke [34], which was in line with this study. Moreover, pre-stroke depression not only increased the risk of stroke, especially in males [35], but also remarkably increased the probability of post-stroke depression (PSD) [36]. These findings implied that higher NIHSS score, Hcy level, and HAMD score played a detrimental role in the development and outcome of BGRI patients in this study.

Accumulating evidence suggested the engagement of gut microbiota in neuropsychological diseases [37,38,39]. One previous study reported dramatically reduced alpha and beta diversities in AIS patients with an unfavorable outcome [40], while another study exhibited high similarities for both alpha and beta diversities between ischemic stroke patients and healthy individuals [13]. Here, our results revealed no significant alteration in alpha diversity but an obvious difference in beta diversity. In this study, the specific genera related to the good-BGRI group, namely, *Romboutsia* and *Fusicatenibacter*, had significant negative correlations with the mRS score. *Romboutsia*, which are capable of generating acetate [41], showed a decreased abundance in both subacute and convalescent phases of stroke [42], implying the continuous influence of *Romboutsia* on the prognosis of stroke. The relative abundance of *Romboutsia* was significantly downregulated in patients with essential tremor and was utilized to efficaciously discriminate patients from healthy individuals [43], suggesting the potent association between *Romboutsia* and motor function. As for *Fusicatenibacter*, it was a subordinate to *Lachnospiraceae*, one of the main butyrate-producing bacteria in human gut [44]. Although *Fusicatenibacter* was not reported to be related to stroke before, the change in its abundance had been observed in other neurological diseases. It was significantly decreased in PD with more intensive inflammation in the intestine [45], which was further identified as a critical biomarker of the progression of PD in another study [46]. In a study of the Thai population, the abundance of *Fusicatenibacter* was dramatically downregulated in dementia patients [47]. According to the evidence above, we assumed that these two genera contributed to the maintenance of gut–brain axis in relatively healthy individuals and had a positive impact on the recovery of BGRI. Intriguingly, most of the changes observed in this study were similar to those observed in PD, which made us suspect that similar mechanisms could be involved in both phenomena, though further study is needed for the validation of this hypothesis.

Additionally, there was an extensive crosstalk between gut microbiota and BGRI-related risk factors in this study. Type 2 diabetes was a well-known risk factor for stroke and had been demonstrated to disrupt gut microbiota homeostasis and exacerbate AIS [48]. In this study, diabetic patients were more likely to show a lower relative abundance of *Romboutsia*, which was also decreased in diabetic retinopathy patients [49]. The beneficial effect of *Romboutsia* was probably attributed to the amended metabolic endotoxemia [50], which was usually caused by type 2 diabetes and played a detrimental role in the severity and outcome of stroke [51]. Consistent with the inverse shift of *Romboutsia* and HAMD score, the same relationship was also observed in PD patients [52]. Contrary to the beneficial effects of *Romboutsia*, *Acetanaerobacterium*, one of the pro-inflammatory genera, was highlighted in the poor-BGRI group and had significant positive associations with CRP as well as hs-CRP. The decreased richness of *Acetanaerobacterium* had been demonstrated to be correlated with an anti-inflammatory effect in enteritis [53]. As elevated uric acid was identified as a risk factor of stroke incidence and progression in both sexes [54] and the changes in uric acid and *UBA1819* in the same direction were revealed in hyperuricemia treatment [55], the *UBA1819* might play a role in the clinical outcome of poor-BGRI patients together with elevated uric acid. By increasing the probability of poor outcome in this study, Hcy showed positive correlations with *UBA1819*, *Staphylococcus*, and *Parascardovia* and negative correlation with *Lachnospiraceae_ND3007_group*, though these relationships were not reported before, and they might be relevant in this study. From the evidence above, we speculated that the interaction of gut microbiota and BGRI-related risks might partially explain the discrepancy in the functional outcome of BGRI.

Notably, *Romboutsia* and *Fusicatenibacter*, which are capable of producing acetate and butyrate, respectively, captured our attention for their common ability to produce SCFAs. SCFAs are generated by fermenting dietary fiber and play a pivotal role in the homeostasis of gut microbiota and overall health [56]. In stroke patients, SCFAs, especially acetate, were negatively associated with the 3-month functional outcome [57]. The beneficial effects of SCFAs in post-stroke neurological deficits and inflammation in aged stroke mice had also been demonstrated [58]. Another study revealed that it was butyrate that mainly contributed to the treatment of stroke by increasing beneficial gut microbiota and improving leaky gut [59]. Furthermore, the supplementation of SCFAs improved impaired limb motor function by modulating microglial activation and recruitment of T cells in an infarcted area [60]. Moreover, the supplementation of SCFAs also alleviated PSD [61] and post-stroke cognitive impairment (PSCI) [62]. In mice with PSCI, the administration of butyrate was capable of repairing ileal epithelial cells and leaky gut, decreasing peripheral inflammatory indices and ameliorating neuronal apoptosis in hippocampus, resulting in the improvement of PSCI [63]. From the perspective of gut–brain axis, we assumed that the discrepancy of prognosis in this study might be partially attributed to richer SCFAs-producing bacteria and subsequently an increased concentration of SCFAs. In addition, ROC curves plotted based on these two SCFAs-producing genera exhibited a strong ability to discriminate good outcome patients from poor outcome patients, indicating the possibility of characteristic microbiota being used in predicting the prognosis of BGRI.

There were several limitations of this study that should be noted. Firstly, the limited number of samples was apparent, and it might aggravate the latent bias. Secondly, we failed to detect metabolites of gut microbiota, and the metagenomics analysis was not conducted and that restricted investigations at the species level. Thirdly, we only collected fecal samples once and did not observe dynamic changes in gut microbiota after the stroke onset. Nevertheless, the results of this study still provided insights into the predictive role of gut microbiota in the prognosis of BGRI, which was not reported before.

## 5. Conclusions

This study revealed a significantly altered gut microbiota in different functional outcomes of BGRI and identified SCFAs-producing bacteria that contributed to the good outcome of BGRI. *Romboutsia* and *Fusicatenibacter* were novel candidate biomarkers for early prediction of the prognosis of BGRI in this study. Though more research is needed, this study still provided robust evidence on the predictive application of gut microbiota in the prognosis of BGRI. 

## Figures and Tables

**Table 1:** Patients’ demographic and clinical characteristics.

Characteristics	Good-BGRI (n = 43)	Poor-BGRI (n = 22)	*p*-Value
Demographic parameters			
Age (years)	63.4 (10.7)	67.1 (13.4)	0.231
Male, n (%)	23 (53.5%)	10 (45.5%)	0.540
Married, n (%)	38 (88.4%)	16 (72.7%)	0.214
Educational level, n (%)			0.742
Illiteracy	9 (20.9%)	3 (13.6%)	
Primary school	18 (41.9%)	11 (50.0%)	
Junior high school or above	16 (37.2%)	8 (36.4%)	
Previous history			
Hyperlipidemia, n (%)	24 (55.8%)	9 (40.9%)	0.255
Diabetes, n (%)	12 (27.9%)	8 (36.4%)	0.485
Hypertension, n (%)	26 (58.1%)	17 (77.3%)	0.127
Hospital examination			
SBP (mmHg)	151.0 (25.0)	167.5 (37.5)	0.364
DBP (mmHg)	88.0 (13.0)	86.5 (22.0)	0.584
MAP (mmHg)	109.8 (12.0)	110.3 (17.3)	0.881
CRP (mg/L)	3.13 (0.32)	3.30 (4.12)	0.158
Hs-CRP (mg/L)	0.94 (1.20)	2.38 (4.90)	0.005
HbA1c (%)	5.88 (1.52)	5.93 (1.85)	0.551
FBG (mmol/L)	5.45 (1.79)	5.11 (1.71)	0.593
Hcy (μmol/L)	9.20 (4.90)	11.75 (5.70)	0.098
TG (mmol/L)	1.70 (1.38)	1.52 (0.82)	0.551
HDL (mmol/L)	1.04 (0.39)	0.99 (0.28)	0.349
TC (mmol/L)	4.67 (0.98)	4.62 (1.01)	0.849
LDL (mmol/L)	3.07 (0.96)	3.04 (0.96)	0.899
Folate (ng/mL)	10.5 (6.6)	8.9 (4.5)	0.181
Vitamin B12 (pg/mL)	350 (152)	339 (313)	0.967
Uric Acid (μmol/L)	313 (81)	320 (83)	0.733
FT3 (pg/mL)	2.99 (0.43)	2.97 (0.44)	0.809
FT4 (ng/dL)	1.17 (0.19)	1.21 (0.18)	0.481
TSH (μIU/mL)	1.906 (1.941)	1.906 (1.079)	0.311
Scale scores			
NIHSS score	2 (2.0)	3 (4.5)	0.001
HAMA score	7 (4.0)	11 (8.5)	0.019
HAMD score	5.0 (4.0)	10.5 (8.0)	0.001
PSQI score	3.0 (2.0)	3.5 (8.0)	0.058
MMSE score	24 (6.0)	24 (8.3)	0.319

**Table 2:** Multivariate logistic regression analysis.

Parameters	B (SE)	*p*-Value	OR	95% CI
Hcy	0.260 (0.119)	0.029	1.297	1.026–1.639
NIHSS score	0.536 (0.270)	0.047	1.710	1.007–2.901
HAMD score	0.553 (0.277)	0.046	1.738	9.010–2.990

## Data Availability

The datasets used during the current study are available from the corresponding author on reasonable request.

## Abbreviations

AIS, acute ischemic stroke; BGRI, basal ganglia region infarction; NIHSS, National Institutes of Health Stroke Scale; SCFAs, short chain fatty acids; mRS, modified Rankin Scale; PD, Parkinson’s disease; MRI, magnetic resonance imaging; SBP, systolic blood pressure; DBP, diastolic blood pressure; MAP, mean arterial pressure; CRP, C-reactive protein; hs-CRP, hypersensitive C-reactive protein; Hcy, homocysteine; TG, triglycerides; TC, total cholesterol; HDL, high-density lipoprotein; LDL, low-density lipoprotein; HbA1c, glycosylated hemoglobin; FBG, fasting blood glucose; FT3, free triiodothyronine; FT4, free tetraiodothyronine; TSH, thyroid stimulating hormone; HAMA, Hamilton Anxiety Scale; HAMD, Hamilton Depression Scale; PSQI, Pittsburgh Sleep Quality Index; MMSE, Mini-Mental State Examination; PCoA, principal coordinate analysis; LDA, linear discriminant analysis; LEfSe, Linear discriminant analysis effect size; ROC, receiver operating characteristic; AUC, area under the curve; ORs, odds ratios; CI, confidence interval; and PSD, post-stroke depression.
